# What makes endolymphatic hydrops MRI work: a visual guide to better protocol choices

**DOI:** 10.1186/s13244-026-02452-9

**Published:** 2026-11-07

**Authors:** Anja Bernaerts, Kathleen Dhont, Shimon Rahman, Filip Deckers, Cathérine Blaivie, Joost van Dinther, Andrzej Zarowski, Floris Wuyts, Bert De Foer

**Affiliations:** 1 Department of Radiology, ZAS Augustinus, Antwerp, Belgium; 2 Faculty of Science, University of Antwerp, Antwerp, Belgium; 3 European Institute for ORL-HNS, Antwerp, Belgium; 4 Lab for Equilibrium Investigations and Aerospace, University of Antwerp, Antwerp, Belgium

**Keywords:** Deep learning reconstruction, Endolymphatic hydrops, High-relaxivity contrast agent, Magnetic resonance imaging, MRI protocol optimization

## Abstract

**Abstract:**

Endolymphatic hydrops MRI is used for the diagnostic evaluation of patients with primary or secondary endolymphatic hydrops. Delayed intravenous post-Gd 3-dimensional fluid-attenuated inversion recovery (3D-FLAIR) and 3-dimensional real inversion recovery (3D-real IR) sequences are the most widely applied techniques, because they exploit subtle Gd-related signal differences between perilymph and endolymph. Yet image quality and diagnostic confidence depend on protocol design, and variation persists between institutions, scanners, and vendors. This educational review provides a visually oriented, practical framework for optimising endolymphatic hydrops MRI, focusing on three core determinants: acquisition parameters, post-processing strategies, and contrast-agent selection. The physical principles underlying key sequence-parameter choices are explained, including the use of long echo trains to maximise acquisition efficiency, prolonged repetition times to enhance the perilymphatic signal, and inversion times tailored to the selected repetition time to achieve effective suppression of the endolymphatic signal. Representative graphs and imaging examples illustrate how protocol modifications influence contrast and diagnostic performance. The review further discusses post-processing approaches and emerging artificial intelligence–based techniques, including deep learning reconstruction, as tools to improve image quality, shorten scan time, or support higher spatial resolution. Finally, the role of gadolinium-based contrast agents, scan delay, and clinical protocol design is also addressed. Four-hour delayed intravenous imaging remains the current standard, although sequence-specific timing, single- or two-step protocols, and half-dose high-relaxivity agents may offer opportunities for tailored imaging, reduced Gd exposure, and improved sustainability. By linking sequence principles to practical protocol choices, this review aims to support reproducible, high-quality hydrops MRI across clinical settings.

**Key Points:**

***Question*** Optimisation and standardisation of 3D-FLAIR and 3D-real IR protocols are essential for accurate, reproducible assessment of endolymphatic hydrops across institutions, scanner platforms, and vendors.

***Findings*** Use TE 500–700 ms and a long echo train for efficiency/background suppression, TR ~ 16,000 ms to enhance perilymph, and TI 2400–3000 ms to suppress endolymph.

***Critical relevance statement*** Emerging deep-learning reconstruction techniques may shorten acquisition times or improve spatial resolution, while half-dose, high-relaxivity GBCAs may provide hydrops visualisation comparable to higher-dose conventional contrast protocols.

**Graphical Abstract:**

**Figure:**
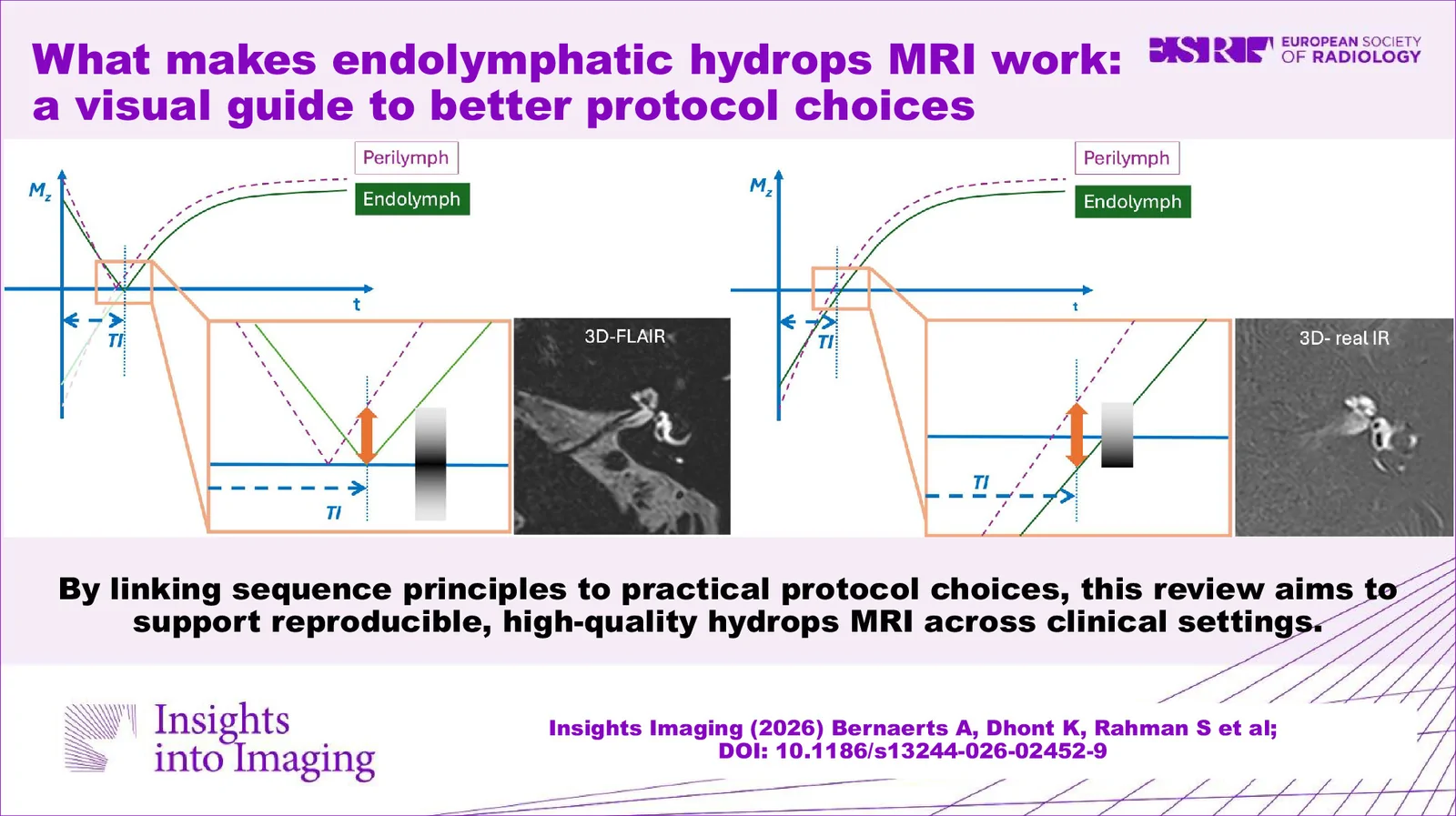

## Introduction

Endolymphatic hydrops (EH) Magnetic Resonance Imaging (MRI) has evolved from a niche research tool into a clinically useful adjunct for assessing suspected hydropic ear disease, particularly Ménière’s disease (MD), atypical or evolving presentations, and selected cases in which imaging may influence treatment decisions. In routine practice, the most defensible standardised approach is currently a 4-h delayed intravenous (IV) 3-T protocol using a high-channel head coil, with a voxel size roughly 0.6 × 0.6 × 1 mm to 0.8 mm isotropic, and either 3-dimensional fluid-attenuated inversion recovery (3D-FLAIR) or three-dimensional inversion recovery with real reconstruction (3D-real IR) as the primary diagnostic sequence [1]. Because EH MRI is designed to depict very small inner-ear fluid spaces and relies on subtle side-to-side signal differences, often requiring relatively long acquisition times, these sequences are more susceptible to patient-motion artefacts than conventional posterior fossa and inner-ear MRI. Careful patient positioning and immobilisation are therefore essential for diagnostic image quality. In practice, inflatable coil-gap pads are often preferable to standard foam pads or wedges, as they provide more stable and individualised head fixation within the coil. Imaging should be accompanied by a side-specific structured report that documents cochlear hydrops, vestibular hydrops, perilymphatic enhancement (PE) asymmetry, vestibular aqueduct visibility and enhancement, overall image quality, and any alternative or coexisting diagnoses [2–6].

A key analytical consideration in EH MRI is that sequence parameterisation directly influences the performance and reliability of imaging descriptors. Acquisition and reporting therefore cannot be treated as separate processes, because the diagnostic value of a descriptor depends on the technical conditions under which it is generated. The 2022 consensus paper emphasised this point, particularly for single-dose IV gadolinium (Gd) protocols, in which even minor deviations in sequence parameters may substantially reduce diagnostic usability [1]. Explicit protocol standardisation is therefore essential in every EH MRI study. The central technical challenge is the very low concentration of Gd that reaches the inner ear after IV administration: only a small amount enters the perilymph, whereas the endolymph remains unenhanced. Consequently, diagnostic performance depends on subtle but critical choices in time to inversion (TI), time to echo (TE), time to repetition (TR), flip-angle evolution and voxel size. This educational review distils lessons from 15 years of clinical practice and protocol-optimisation research since the introduction of EH MRI in 2010, focusing on sequence parameterisation, post-processing, and contrast administration. Standardisation and optimisation of these interdependent components are critical for reliable interpretation, reproducible measurements, and meaningful comparison across studies. Because implementation differs across vendors, field strengths, coils, and reconstruction platforms, the protocol values provided here should be regarded as validated starting points rather than universal settings.

## Sequence families: basic principles and their strengths

**Fig. 1:**
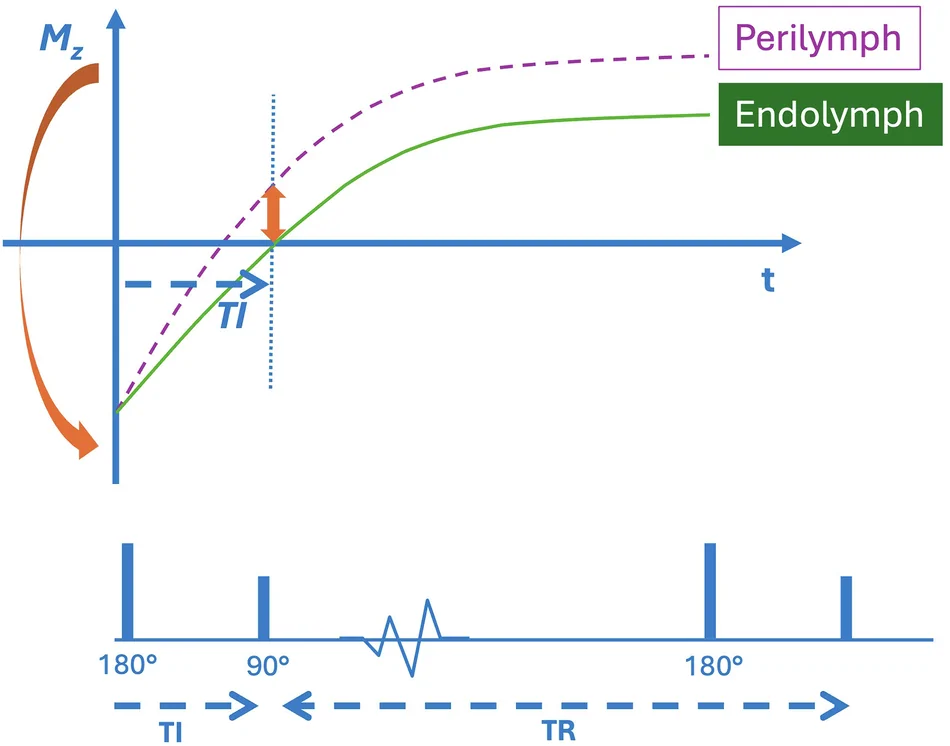
Principle of TI selection in IR hydrops MRI. Illustration of the temporal evolution of longitudinal magnetisation (Mz) in an IR sequence. After a 180° inversion pulse, endolymph and Gd-enhanced perilymph recover Mz at different rates, with enhanced perilymph recovering more rapidly because of T1 shortening. At the selected TI, a 90° excitation pulse is applied, and the resulting transverse magnetisation (Mxy) is measured. By setting the TI at the endolymph null point, endolymph produces no Mxy and appears suppressed, whereas enhanced perilymph retains signal, thereby maximising endolymph–perilymph contrast (vertical double arrow). The graph does not depict the T1 relaxation curve after the 90° excitation pulse

When Gd is administered IV, only a small amount crosses the blood-perilymph barrier. Because of this, standard spin echo T1 imaging cannot detect the low levels present in the perilymph and does not offer enough contrast to distinguish endolymph from perilymph. To overcome this limitation, an inversion recovery (IR) sequence can be used, which doubles sensitivity to T1 differences by adding a 180-degree inversion pulse—providing twice the dynamic range compared to a standard T1-weighted spin echo pulse sequence. Moreover, the contrast between the slightly enhanced perilymph and endolymph can be further increased by selectively suppressing or nulling the signal from endolymph using an appropriate TI (Fig. 1).

In hydrops imaging, two main IR sequences are commonly used, each utilising a distinct reconstruction method. FLAIR sequences utilise magnitude reconstruction, while real-IR employs phase-sensitive reconstruction. The following paragraphs will elucidate these differences in greater detail. In current practice, delayed 3D-FLAIR is the dominant routine sequence, while 3D-real IR and HYDROPS-family reconstructions are used when more explicit separation of endolymph, perilymph, and bone is needed, or when volumetric assessment is desired.

### 3D-FLAIR sequence

**Fig. 2:**
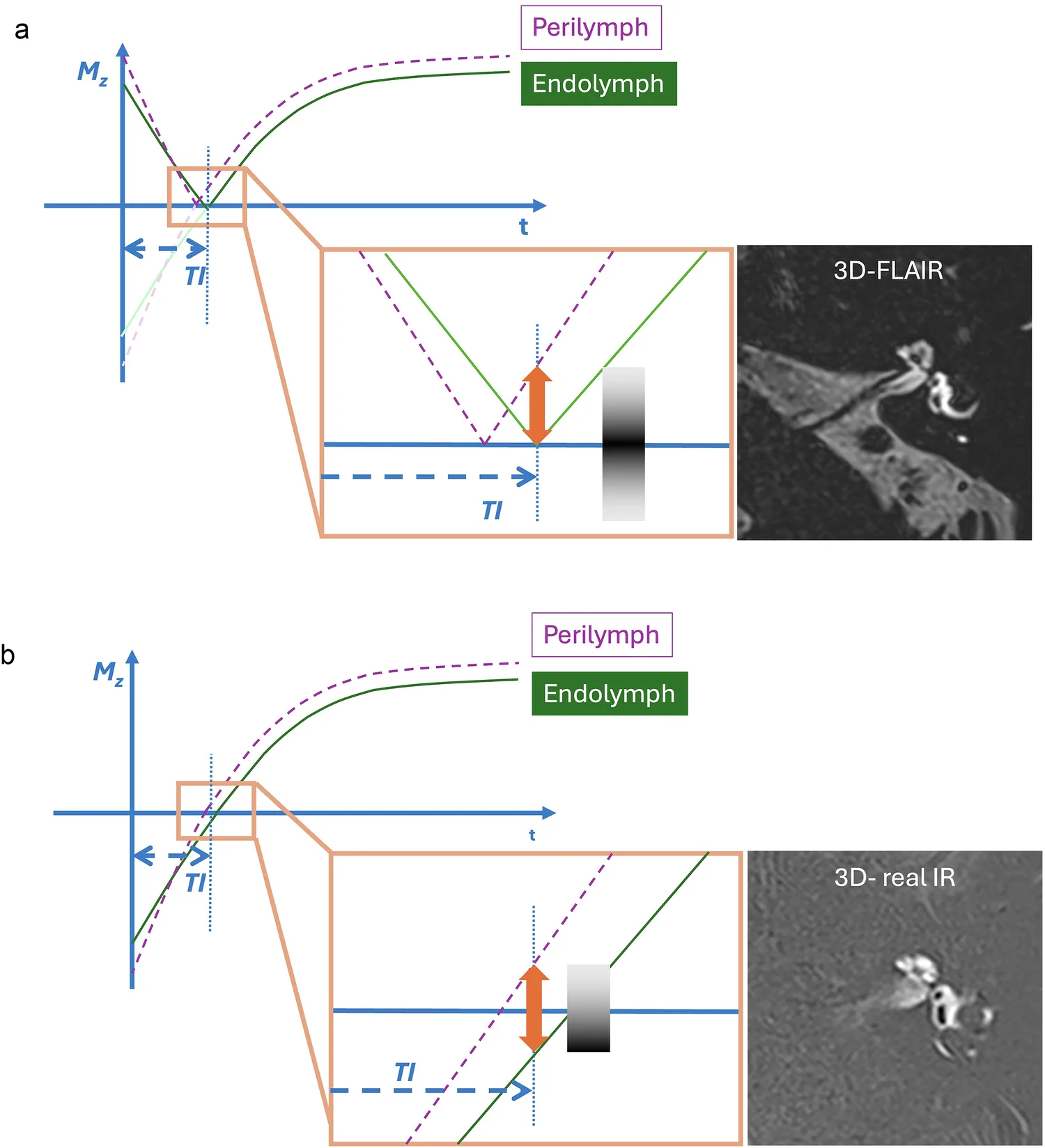
Principles of magnitude (3D-FLAIR) and phase-sensitive (3D-real IR) IR reconstruction in hydrops MRI. Two main IR sequences are used for delayed post-Gd hydrops imaging, differing primarily in their reconstruction approach: 3D-FLAIR uses magnitude reconstruction, whereas 3D-real IR uses phase-sensitive reconstruction. **a** Magnitude reconstruction in 3D FLAIR. The graph illustrates the evolution of Mz after an inversion pulse. With magnitude reconstruction, only the absolute signal magnitude is displayed, while signal polarity is discarded; therefore, positive and negative magnetisation are represented equivalently. The TI is selected at the endolymph null point. At the time of the 90° excitation pulse, endolymph has no Mz to be converted into Mxy and therefore appears black, whereas Gd-enhanced perilymph generates signal and appears bright. This principle forms the basis of conventional 3D-FLAIR hydrops imaging. **b** Phase-sensitive reconstruction in 3D real IR. The graph illustrates phase-sensitive reconstruction, in which both the magnitude and polarity of Mz are preserved. The TI is selected between the null points of endolymph and perilymph, thereby generating distinct signal polarities and improving tissue separation. As a result, negative dark endolymphatic signal and positive bright perilymphatic signal can be distinguished from the intermediate grey signal of the surrounding bone in a single image, without the need for mathematical subtraction. This principle underlies single-TI 3D-real IR imaging

FLAIR is an IR sequence reconstructed using magnitude data, such that signal intensity reflects the absolute magnitude of the longitudinal magnetisation (Mz), irrespective of its polarity. The TI is selected to coincide with the zero-crossing point of non-enhancing endolymph. At this time, the Mz of the endolymph is effectively zero; consequently, the subsequent excitation pulse generates little or no transverse magnetisation (Mxy) and therefore no detectable signal. Endolymph thus appears dark, whereas Gd-enhanced perilymph, which has recovered beyond its zero-crossing point because of its shorter T1 relaxation time, appears bright (Fig. 2a).

The main weakness of this sequence is that endolymph and surrounding bone are both dark, which makes anatomical delineation harder, especially when the perilymphatic rim is lost [7, 8].

### 3D-real IR sequence

A phase-sensitive (or real) reconstruction solves that contrast problem by preserving signal polarity of the Mz, with negative values being of increasingly lower signal. With a shorter TI set midway between the zero-crossing points of endolymphatic and perilymphatic signal, a phase-sensitive reconstruction will depict negative endolymphatic signal and positive perilymphatic signal separately from that of the grey signal of the bony labyrinth without mathematical subtraction in the same single image (Fig. 2b).

**Fig. 3:**
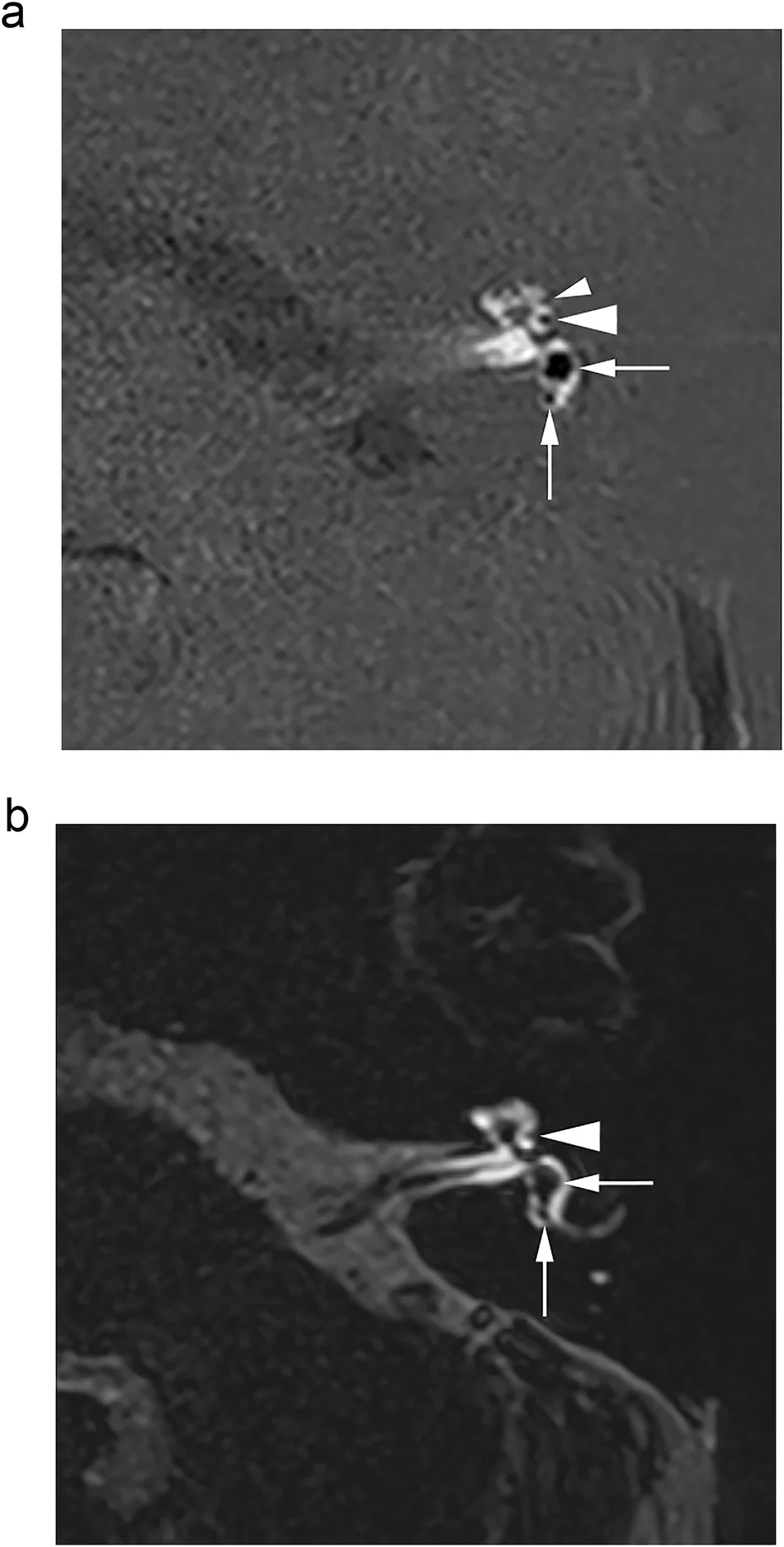
Improved visualisation of subtle cochlear EH on 3D-real IR. In clinical practice, 3D-real IR sequences can be recognised by the characteristic intermediate grey signal of the surrounding bone, in contrast to the black bone signal seen on 3D-FLAIR images. This grey bone background improves visualisation of the black endolymphatic space, particularly in the mid and apical cochlear turns. This patient had long-standing, clinically stable left-sided Ménière disease and presented with a recent vertigo episode 3 weeks earlier. Delayed post-Gd MRI demonstrates grade 1 vestibular and cochlear EH according to the Bernaerts grading system. Vestibular hydrops is shown by an enlarged saccule (horizontal arrow), which is larger than the utricle (vertical arrow) but not confluent with it. Cochlear hydrops is seen as nodular enlargement of the cochlear duct. On the 3D-real IR image (**a**), nodular distension of the cochlear duct is clearly visible in both the mid turn (large arrowhead) and apical turn (small arrowhead) against the grey bone background. On the corresponding 3D-FLAIR image (**b**), the abnormality is less conspicuous because the bone background is also black, reducing contrast with the endolymphatic space; only the nodularly distended cochlear duct in the mid turn is readily appreciated (large arrowhead)

In clinical application, real-IR sequences can be identified by the distinctive intermediate grey signal of the surrounding bone, which stands in contrast to the black signal observed in FLAIR sequences. This feature allows improved visualisation of the small black endolymphatic space in the mid and apical turns, set against the grey background of the surrounding bone (Fig. 3) [7, 8]. However, 3D-real IR may be less sensitive for detecting asymmetric PE [9]. In addition, protocol-dependent longer 3D-real IR acquisitions may increase vulnerability to motion artefacts [8].

### Subtraction-based methods

Subtraction-based methods involve acquiring two 3D-FLAIR sequences with varying TI values. Subtracting the so-called positive endolymph image obtained with a shorter TI from the positive perilymph image acquired with a longer TI effectively distinguishes high-signal perilymph and low-signal endolymph from intermediate-signal bone. This technique is known as HYDROPS (hybrid of reversed image of positive endolymph signal and native image of positive perilymph signal) [10]. HYDROPS-Mi2 multiplies this subtraction image by MR cisternography to greatly increase contrast-to-noise ratio (CNR) [11]. These methods are particularly useful in centres with strong post-processing capabilities but limited access to robust real-reconstruction sequences, as image registration may be necessary to correct for interscan patient motion and prevent subtraction artefacts [12].

## Imaging parameter optimisation for enhanced contrast among perilymph, endolymph, and background

Over the past decade and a half, optimisation of 3D fast spin-echo IR sequence parameters has markedly improved visualisation of the endolymphatic and perilymphatic spaces. In particular, longer TE and TR settings, together with optimised inversion timing, increase perilymphatic signal conspicuity and improve contrast between enhanced perilymph, unenhanced endolymph, and adjacent structures, thereby supporting more reliable diagnosis of EH.

### Long echo-train length and echo time

**Fig. 4:**
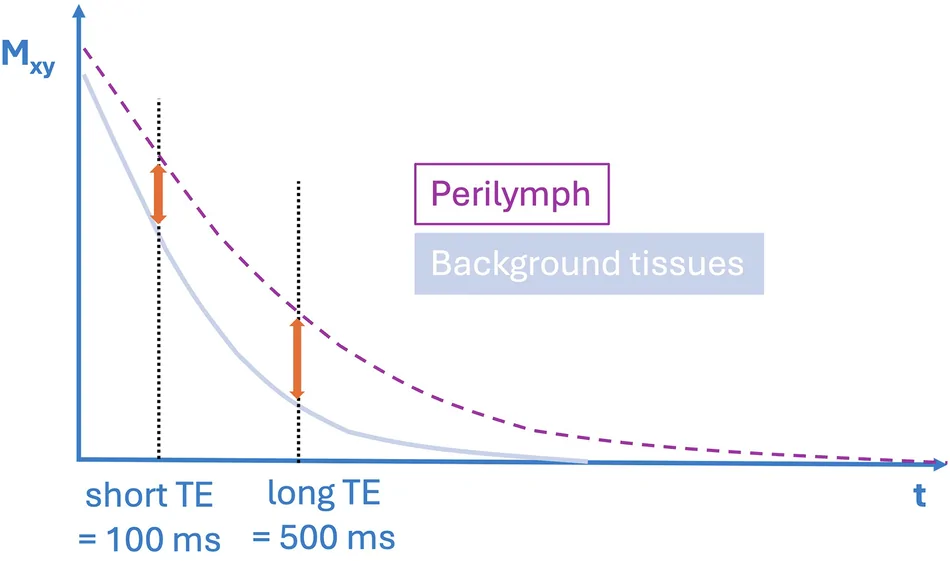
Principle of long-TE heavily T2-weighted IR for improved perilymph-to-background contrast. This graph illustrates how a heavily T2-weighted IR sequence with a long TE improves the contrast between enhancing perilymph and background tissues, including the otic capsule and brain parenchyma (vertical double arrow). The graph shows the temporal evolution of Mxy during an IR sequence. By using a longer TE, for example 500 ms instead of 100 ms, Mxy from background tissues has more time to decay, whereas signal from labyrinthine fluids is relatively preserved because of their exceptionally long T2 relaxation time. Endolymph contributes little or no transverse signal at readout because its Mz is nulled by the selected inversion time (TI), as illustrated in Fig. 1

First, studies have shown that heavily T2-weighted 3D-FLAIR (hT2w-3D-FLAIR) improves the CNR of the perilymph [13]. Owing to their exceptionally long T2 relaxation times, the Mxy of both the endolymph and perilymph decays relatively slowly following radiofrequency excitation and therefore remains comparatively well preserved during the echo train. A long echo train enables multiple k-space lines to be acquired after each excitation, thereby increasing scanning efficiency. In parallel, a prolonged effective TE allows greater decay of Mxy in background tissues while preserving signal from the long-T2 labyrinthine fluids, resulting in enhanced fluid-to-background contrast (Fig. 4).

**Fig. 5:**
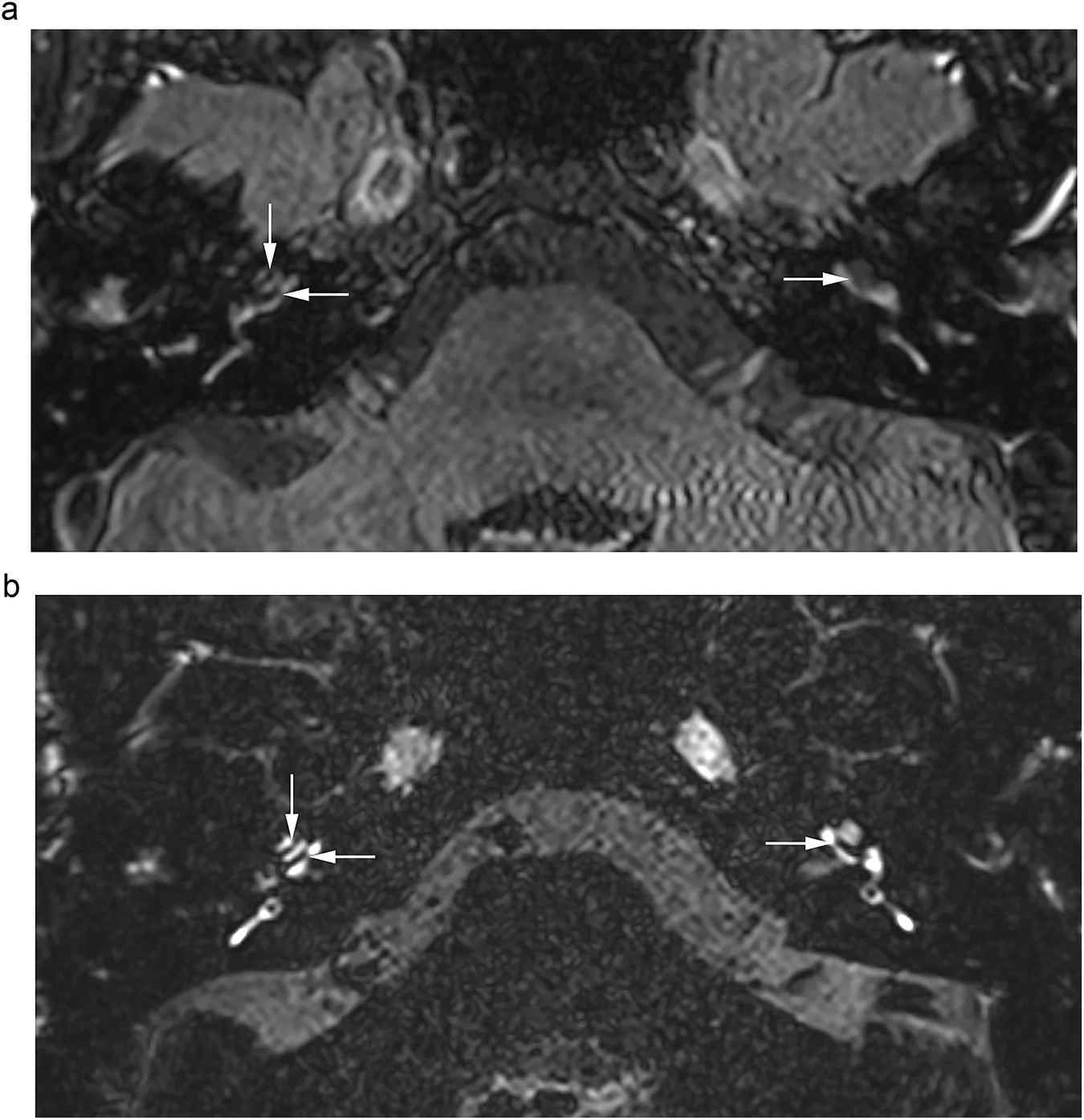
Improved scanning efficiency and perilymph–to-background contrast with long-echo-train, long-TE 3D SPACE FLAIR. A 43-year-old woman with a 2-year history of vertigo, right-sided hearing loss, tinnitus, and aural fullness was clinically diagnosed with definite MD. Imaging was performed 4 h after IV administration of a single Gd dose. **a** Axial 3D TSE FLAIR image (TE, 146 ms) obtained at the level of the basal and middle cochlear turns demonstrates a band-like hypointense signal in the scala vestibuli of the right middle cochlear turn (vertical arrow), consistent with grade 2 cochlear EH according to the Bernaerts grading system. Care should be taken not to misinterpret the normal bony interscalar septum between the basal and middle cochlear turns (horizontal arrows) as a distended cochlear duct. **b** Axial 3D SPACE FLAIR image (TE, 549 ms) obtained at the same level as in (**a**), shows markedly improved delineation of the endolymphatic and perilymphatic spaces and perilymph-to-background contrast compared with 3D TSE FLAIR. The band-like enlargement of the cochlear duct in the right middle cochlear turn, consistent with grade 2 cochlear EH, is more conspicuous (vertical arrow). Improved background suppression also provides clearer visualisation of the normal bony interscalar septum between the basal and middle cochlear turns (horizontal arrows)

Three-dimensional sampling perfection with application-optimised contrasts using different flip-angle evolutions (3D SPACE) FLAIR is an example of an hT2w-3D-FLAIR sequence and provides spatial resolution comparable to that of conventional 3D TSE FLAIR. Its extended echo-train length and prolonged effective TE of approximately 500–700 ms have been shown to improve sensitivity for the visualisation of EH (Fig. 5) [14]. The highest diagnostic performance and CNR have been reported with a constant rather than variable refocusing flip-angle scheme. Among the evaluated constant flip angles, 140° outperformed 120°, likely because the higher refocusing angle reduces signal loss over the prolonged echo train [15, 16].

### Long repetition time

**Fig. 6:**
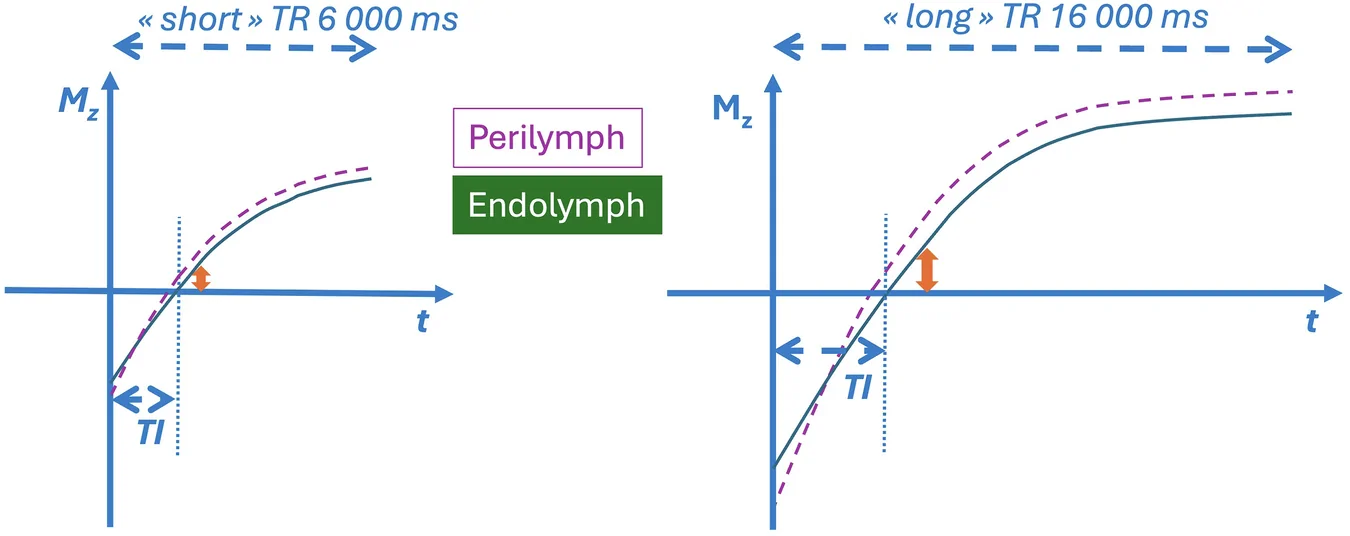
Principle of long-TR IR for improved perilymph–endolymph contrast. These graphs show how prolonging the repetition time (TR) can improve contrast between Gd-enhanced perilymph and endolymph in IR hydrops MRI. With a shorter TR, such as 6000 ms, Mz shows only limited recovery before the next inversion pulse, resulting in reduced separation between the endolymphatic and perilymphatic signals and therefore limited contrast (vertical double arrow). In contrast, a longer TR, such as 16,000 ms, allows greater recovery of Mz, increasing the difference between the two fluid compartments at the time of readout. This improves qualitative visualisation of the endolymphatic and perilymphatic spaces (vertical double arrow). The TI must be adapted to the selected TR to maintain appropriate suppression of the endolymphatic signal

**Fig. 7:**
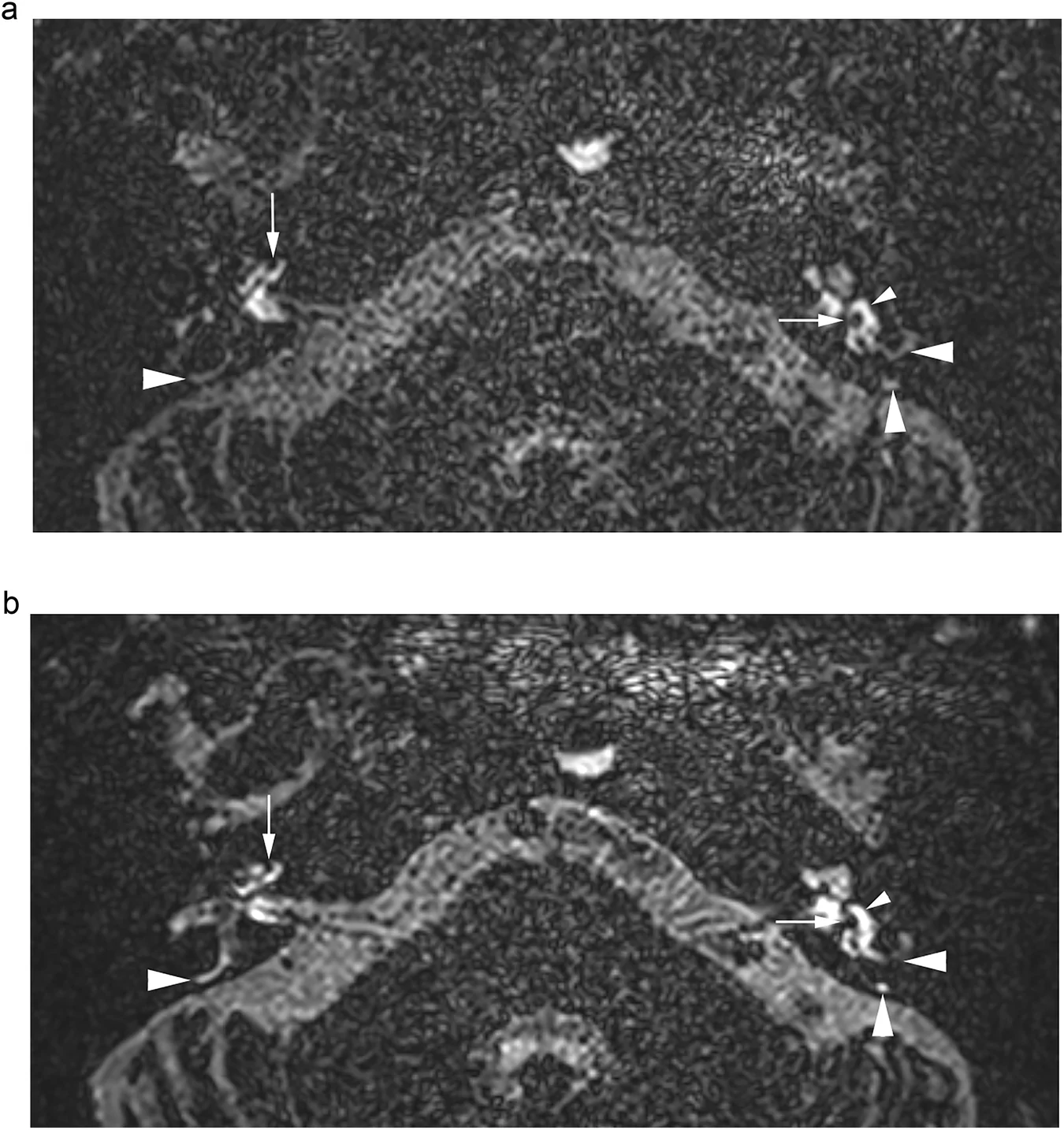
Enhanced perilymphatic signal on long-TR 3D SPACE FLAIR. 3D SPACE FLAIR images in a 67-year-old man with bilateral Ménière disease, acquired with a shorter TR of 10,000 ms (**a**) and a longer TR of 16,000 ms (**b**) at the level of the inferior vestibule and middle cochlear turn. The images demonstrate grade 1 vestibular EH on the left according to the Bernaerts grading system, characterised by a saccule larger than the utricle without confluence (horizontal arrow), and grade 1 cochlear EH on the right, characterised by nodular dilation of the cochlear duct (vertical arrow). The longer-TR acquisition (**b**) provides increased perilymphatic signal, improving contrast in the vestibule (small arrowhead) and enhancing visualisation of the smaller-calibre semicircular canals (large arrowheads)

Secondly, an extended TR in the 3D-FLAIR sequence has demonstrated advantages by enabling adequate recovery of Mz, particularly given the weak T1 shortening associated with low Gd concentrations [17–21]. Employing a longer TR of 16,000 ms permits more Mz recovery, thereby enhancing contrast between endolymph and perilymph. Conversely, a shorter TR (e.g. 6000 ms) allows only limited Mz recovery, resulting in reduced contrast between these two fluids (Fig. 6). This increased CNR between perilymph and endolymph contributes to improved qualitative visualisation of the inner ear fluid compartments (Fig. 7).

It is essential to note that, under these conditions, the TI (2400–3000 ms) must be adjusted according to the chosen TR to ensure effective suppression of the endolymphatic signal. Additionally, while extended TR values can improve contrast, they adversely lead to longer scan times.

### State-of-the-art acquisition parameter recommendations

Detailed numerical parameter recommendations are currently best supported for Siemens platforms, reflecting the predominance of Siemens-based phantom and clinical optimisation studies rather than intrinsic vendor superiority. Although the underlying optimisation principles are broadly applicable, exact settings require vendor- and platform-specific adaptation and validation. Current state-of-the-art Siemens parameters are summarised in Table 1.

**Table 1:** Acquisition parameter recommendations for 3 T MAGNETOM Vida (XA60)

Standard resolution (no deep resolve)		
Sequence	3D SPACE FLAIR	3D SPACE real IR
Orientation	Transversal	Transversal
TR (ms)	16,000	16,000
TE (ms)	551	551
TI (ms)	3100	2700
FOV (mm^2^)	160 × 160	160 × 160
Acquisition Matrix	248 × 256	248 × 256
Number of slices	26	30
Acquired slice thickness (mm)	0,94	1,00
Reconstructed voxel size (mm^3^)	0.31 × 0.31 × 0.80	0.31 × 0.31 × 0.80
Averages	2	2
Parallel imaging	GRAPPA, factor 2	GRAPPA, factor 2
RF bandwidth (Hz/pixel)	501	501
Turbo factor	235	235
Echo train duration (ms)	1113	1113
Flip angle mode	Constant 120°	Constant 120°
Reconstruction	Magnitude	Real
Acquisition time	7 min 12 s	9 min 4 s

Ultra-high resolution (with deep resolve)		
Sequence	3D SPACE FLAIR	3D SPACE real IR
Orientation	Transversal	Transversal
TR (ms)	16,000	16,000
TE (ms)	551	547
TI (ms)	3100	2700
FOV (mm^2^)	160 × 160	160 × 160
Acquisition Matrix	295 × 304	298 × 304
Number of slices	36	36
Acquired slice thickness (mm)	0,77	0,77
Reconstructed voxel size (mm^3^)	0.26 × 0.26 × 0.60	0.26 × 0.26 × 0.60
Averages	2	2
Parallel imaging	GRAPPA, factor 3	GRAPPA, factor 3
RF bandwidth (Hz/pixel)	498	498
Turbo Factor	235	232
Echo train duration (ms)	1158	1128
Flip Angle Mode	Constant 120°	Constant 120°
Reconstruction	Magnitude	Real
Acquisition Time	8 min 50 s	9 min 38 s

Current state-of-the-art acquisition parameters for delayed IV EH imaging using 3D-SPACE FLAIR and 3D-SPACE real-IR sequences, with and without Deep Resolve reconstruction, on a 3-T Siemens MAGNETOM Vida system (software version XA60)

## New developments in image reconstruction techniques for faster and ultra-high-resolution imaging

Hydrops imaging sequences have traditionally required long acquisition times, often up to 14 min, limiting routine clinical use. The main concern is not simply throughput, but preservation of image quality in the small and complex membranous labyrinth, which requires high spatial resolution and strong endolymph–perilymph contrast. Prolonged acquisitions increase motion, swallowing, and body-shift artefacts, particularly in HYDROPS-family methods, where misregistration between mathematically combined source images can create false interfaces or blurred compartment boundaries [22]. This limitation is being addressed by implementing parallel imaging (PI) acceleration, either alone or in combination with iterative denoising, or compressed sensing (CS). Newer acceleration techniques include zoomed-FOV (ZOOMit), T2-preparation, and deep-learning reconstruction (DLR) [22–28].

### PI acceleration

PI reduces acquisition time by undersampling k-space, typically along the phase-encoding direction, and reconstructing the omitted data using spatial information from multichannel receiver-coil sensitivity profiles. In hydrops imaging, commonly used vendor implementations include GRAPPA-type techniques on Siemens systems, SENSE on Philips systems, ASSET on GE HealthCare systems, and SPEEDER on Canon Medical Systems scanners.

The main limitation of PI is reduced signal-to-noise ratio (SNR), with additional risks of geometry factor (g-factor) noise amplification, residual aliasing, or reconstruction failure when coil geometry is unfavourable or acceleration is too high. Therefore, EH MRI has generally favoured moderate acceleration (around 2) and uses high-quality multichannel coils, since the small target structures tolerate little residual reconstruction noise. Coil configuration is therefore crucial: recent studies use 20-, 32-, and 64-channel head or head/neck coils. While more channels don’t guarantee better hydrops images, they generally improve PI performance and reduce g-factor penalties, allowing more flexibility in scan time and matrix adjustments [29].

### New deep learning reconstruction (DLR) technology

In modern MRI systems, these traditional PI and CS methodologies can be combined with DLR for EH imaging by incorporating prior knowledge from training datasets to fill in missing information. DLR can fulfil four distinct roles: denoising, sharpening, artefact reduction, and acceleration. The commercial offerings in DLR technology are considerably more developed than the EH-specific academic literature, as exemplified by platforms such as AIR Recon DL (GE HealthCare), Deep Resolve (Siemens Healthineers), SmartSpeed (Philips), and Advanced intelligent Clear-IQ Engine (AiCE; Canon Medical Systems). Nevertheless, direct EH-specific clinical evidence supporting DLR remains limited and narrow, with only two main Canon/AiCE-related publications about DLR-hydrops from the Nagoya group published so far [22, 23].

Naganawa et al [21] conducted the most robust direct quantitative study to date. In 3T-iHYDROPS-Mi2 imaging, the CNR between endolymph and perilymph improved significantly from 1681.8 ± 845.2 without AiCE to 7738.6 ± 5149.2 with AiCE (*p* = 0.00002), while both endolymph ratio and visual EH grading remained consistent. This substantial gain in image quality is noteworthy [22]. A subsequent technical note by Naganawa et al [22] introduced a rapid HYDROPS2-Mi2 + DLR protocol, reducing acquisition time to approximately 5 min. This method delivered comparable image quality and fewer motion artefacts relative to longer protocols, marking a meaningful advancement for workflow efficiency [23].

**Fig. 8:**
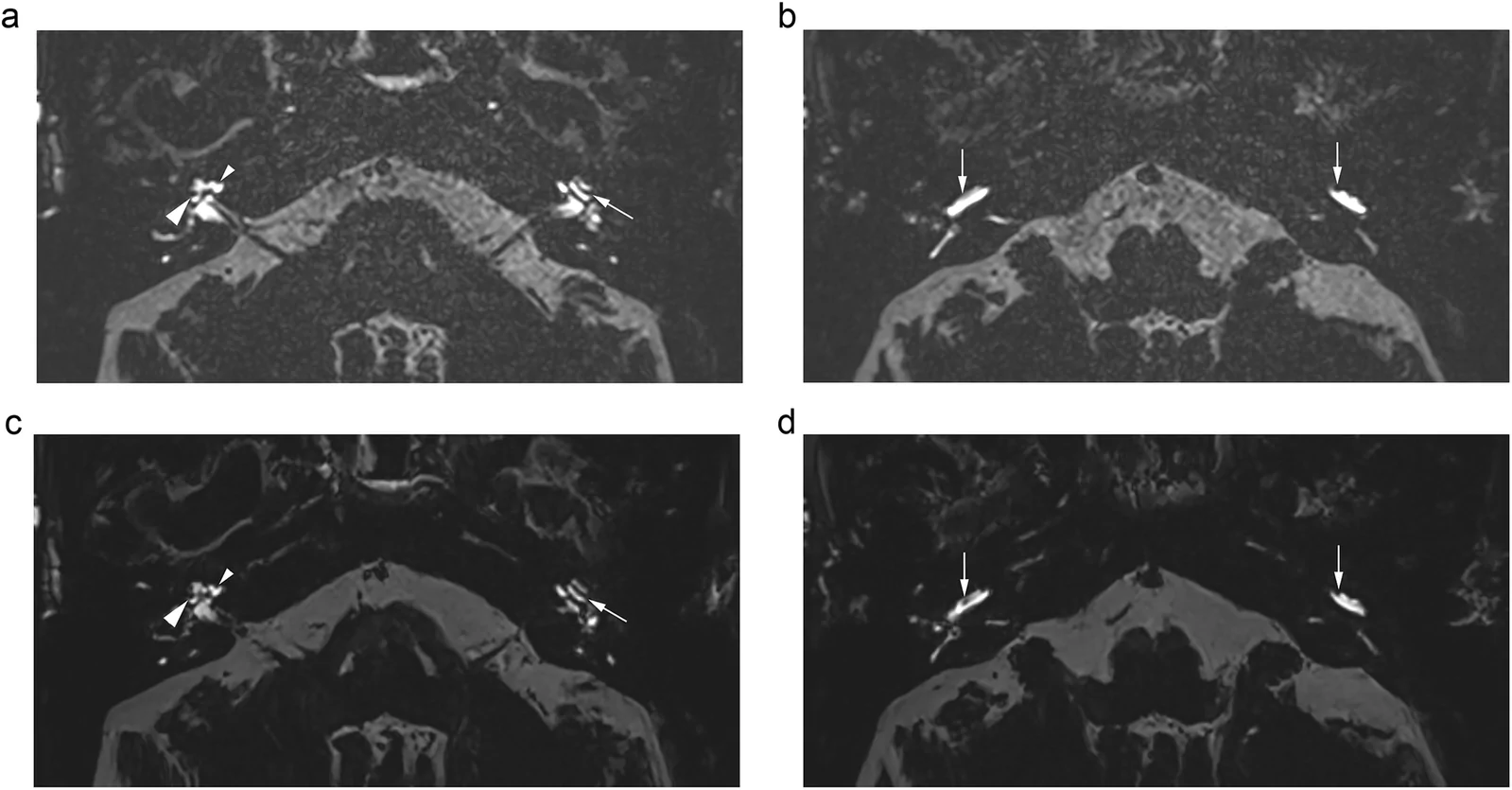
Ultra-high-resolution hydrops MRI with DLR reveals focal cochlear EH. 3D-SPACE FLAIR images acquired at standard resolution (0.6 × 0.6 × 1 mm) (**a**, **b**) and ultra-high-resolution 3D-SPACE FLAIR images reconstructed with Deep Resolve Boost (DRB; 0.3 × 0.3 × 0.6 mm) (**c**, **d**) in a patient with bilateral primary hydropic ear disease. Images are shown at the level of the middle and apical cochlear turns (**a**, **c**) and at the level of the basal cochlear turn (**b**, **d**). At the level of the middle and apical turns (**a**, **c**), band-like enlargement of the cochlear duct is visible in the left cochlea on both acquisitions (horizontal arrow). In the right cochlea, however, DRB (**c**) improves visualisation of variable cochlear duct distension, which is more pronounced laterally in the middle turn (large arrowhead) than medially (small arrowhead) (**c**), compared with the corresponding non-DRB image (**a**). At the level of the basal cochlear turn (**b**, **d**), small discontinuous nodular areas of cochlear duct enlargement are present bilaterally (vertical arrows) and are more clearly delineated with DRB (**d**) than without DRB (**b**). These findings are compatible with focal cochlear hydrops, in keeping with histopathological observations reported in selected cases

Although no EH-specific DLR studies have yet directly compared diagnostic performance metrics—such as sensitivity, specificity, or area under the curve (AUC)—between conventional PI/CS reconstruction and DLR using identical inner-ear datasets, DLR remains a promising approach. By enabling higher reconstructed spatial resolution, with voxel sizes as small as 0.3 × 0.3 × 0.6 mm, DLR may improve the detection of subtle EH, particularly mild or focal cochlear hydrops, in line with histopathological observations [30, 31]. This potential benefit is likely most relevant in the cochlear apex, where PE is often weakest, and the anatomic structures are smallest, making evaluation particularly challenging (Fig. 8). Moreover, high-resolution 3-T 3D-T2-weighted MRI with DLR has already been shown to significantly improve visualisation of the utricle, utricular macula, and ampullary nerves compared with conventional imaging [32].

**Fig. 9:**
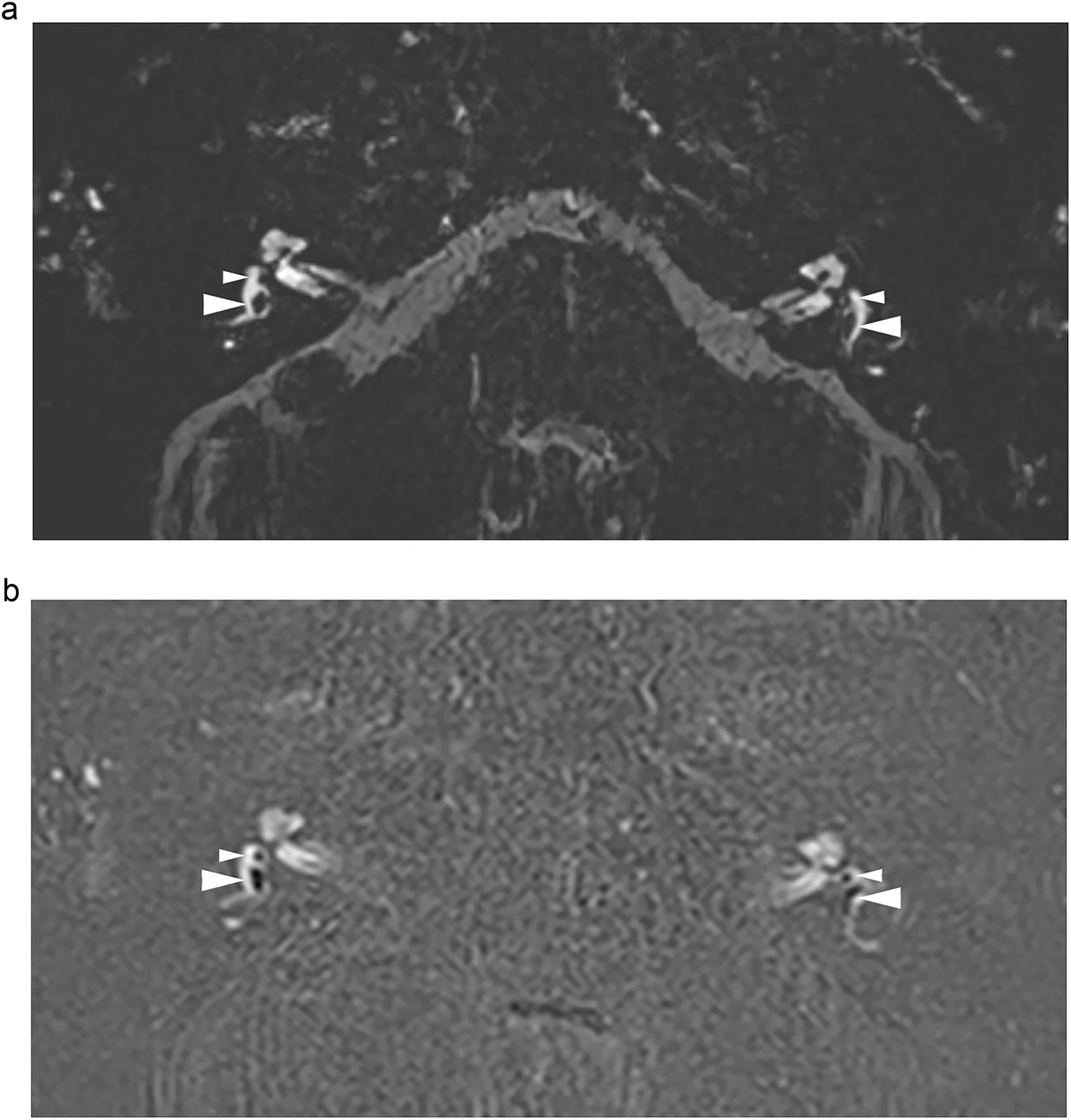
Advancing 1.5-T hydrops MRI toward 3-T-like image quality using DLR. A 40-year-old woman with vertigo, tinnitus, subjective left-sided hearing loss, and a normal audiogram underwent hydrops MRI because the clinical distinction between vestibular migraine (VM) and MD was challenging. Owing to an implant approved only for 1.5-T MRI, imaging was performed at 1.5 T, 4 h after IV administration of a double dose of gadobutrol. 3D SPACE FLAIR (**a**) and 3D-real IR (**b**) sequences were acquired with Deep Resolve Boost (DRB), with acquisition times of approximately 8 min. The images demonstrate symmetric PE, thin, barely visible cochlear ducts bilaterally, and separately identifiable saccules and utricles, with the saccule (small arrowhead) smaller than the utricle (large arrowhead). These findings supported the diagnosis of VM rather than MD. The case illustrates how DLR can improve 1.5-T hydrops MRI image quality to a level approaching that typically associated with 3-T imaging

Current clinical practice generally favours 3-T over 1.5 T MRI for EH imaging because of its higher SNR. This does not imply that 1.5-T MRI is unusable, particularly when combined with a double-dose contrast protocol. Rather, the main trade-off lies in reduced diagnostic confidence for subtle disease, owing to the longer acquisition burden, increased motion sensitivity, and lower perilymphatic SNR at 1.5 T [33]. However, with DLR, shorter and more clinically feasible 1.5-T protocols may become increasingly realistic (Fig. 9).

Yet a major caveat remains: excessive acceleration may compromise image integrity. For EH assessment, DLR must not only reduce noise but also preserve subtle morphology, including saccular configuration, asymmetric PE, scala relationships, and vestibular aqueduct visibility. Images that appear sharper but distort these descriptors may be harmful. Thus, the goal is not simply better-looking images, but more stable and validated EH descriptors.

## Application of GBCA to augment perilymphatic signal

The current clinical default for inner-ear MRI to visualise perilymph–endolymph contrast is delayed IV administration of a single standard dose (0.1 mmol/kg) of a preferably macrocyclic Gd-based contrast agent (GBCA), followed by imaging at about 4 h. Compared with intratympanic administration, this approach is bilateral, less invasive, and better aligned with current safety and regulatory practice, although the weaker perilymphatic signal requires careful sequence optimisation and local validation [1].

**Fig. 10:**
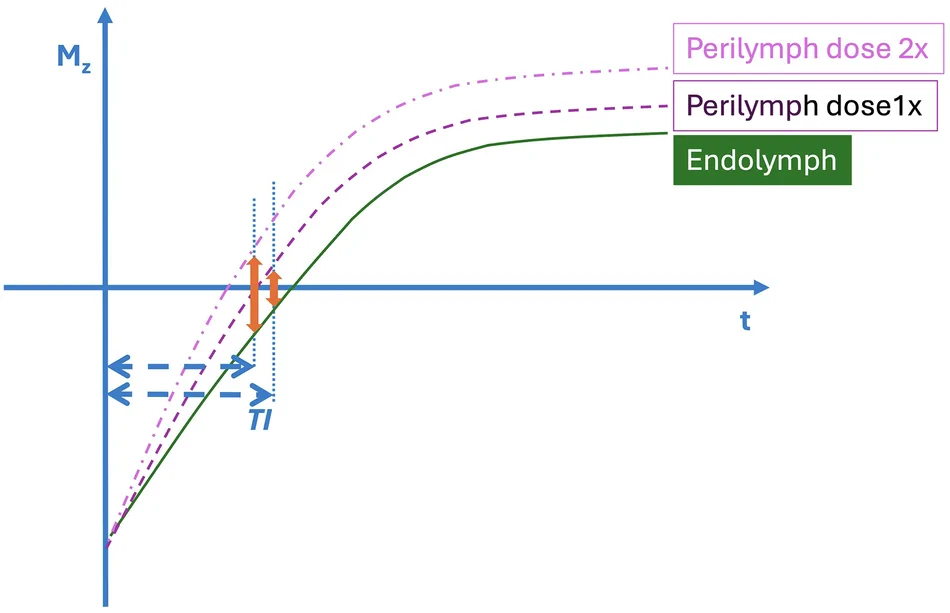
Effect of Gd dose on endolymph–perilymph contrast in IR hydrops MRI. Schematic illustration of the temporal evolution of Mz in an IR sequence for endolymph and perilymph after IV administration of either a single or double dose of GBCA. Compared with a single dose, a double dose induces greater T1 shortening of the perilymph, resulting in faster recovery of Mz and higher perilymphatic signal at readout. A similar effect may be achieved with higher GBCA concentrations or agents with higher relaxivity. At the selected TI, positioned between the null points of endolymph and perilymph as used for phase-sensitive reconstruction in 3D-real IR, the increased perilymphatic recovery enhances the signal difference between the dark endolymphatic compartment and the bright Gd-enhanced perilymph (vertical double arrow). This improves endolymph–perilymph contrast

Perilymphatic Gd enhancement is dose-dependent. A higher administered dose shortens the perilymphatic T1 and accelerates Mz recovery, thereby increasing contrast between the enhancing perilymph and the non-enhancing endolymph (Fig. 10). Separate visualisation of the inner-ear fluid compartments is feasible after a standard single-dose injection [34] but image conspicuity—particularly on 3D-real IR acquisitions—may improve with 1.5-fold or double-dose protocols [35]. However, although double-dose IV protocols (0.2 mmol/kg) can increase perilymphatic signal in less optimised acquisitions, their limited regulatory approval in some countries, together with concerns about Gd deposition, makes them less suitable for repeated imaging.

### High-concentration and high-relaxivity GBCAs

Use of GBCAs with higher molar concentration and/or greater longitudinal relaxivity may enhance PE on delayed inner-ear MRI. Compared with gadoterate meglumine (0.5 mmol/mL), gadobutrol (1.0 mmol/mL) yields higher perilymphatic signal-intensity ratios and improves qualitative visualisation of inner-ear PE. This effect may partly reflect its higher Gd concentration and longitudinal relaxivity, although agent-specific physicochemical properties influencing passage across the blood–perilymph barrier may also contribute [36, 37].

**Fig. 11:**
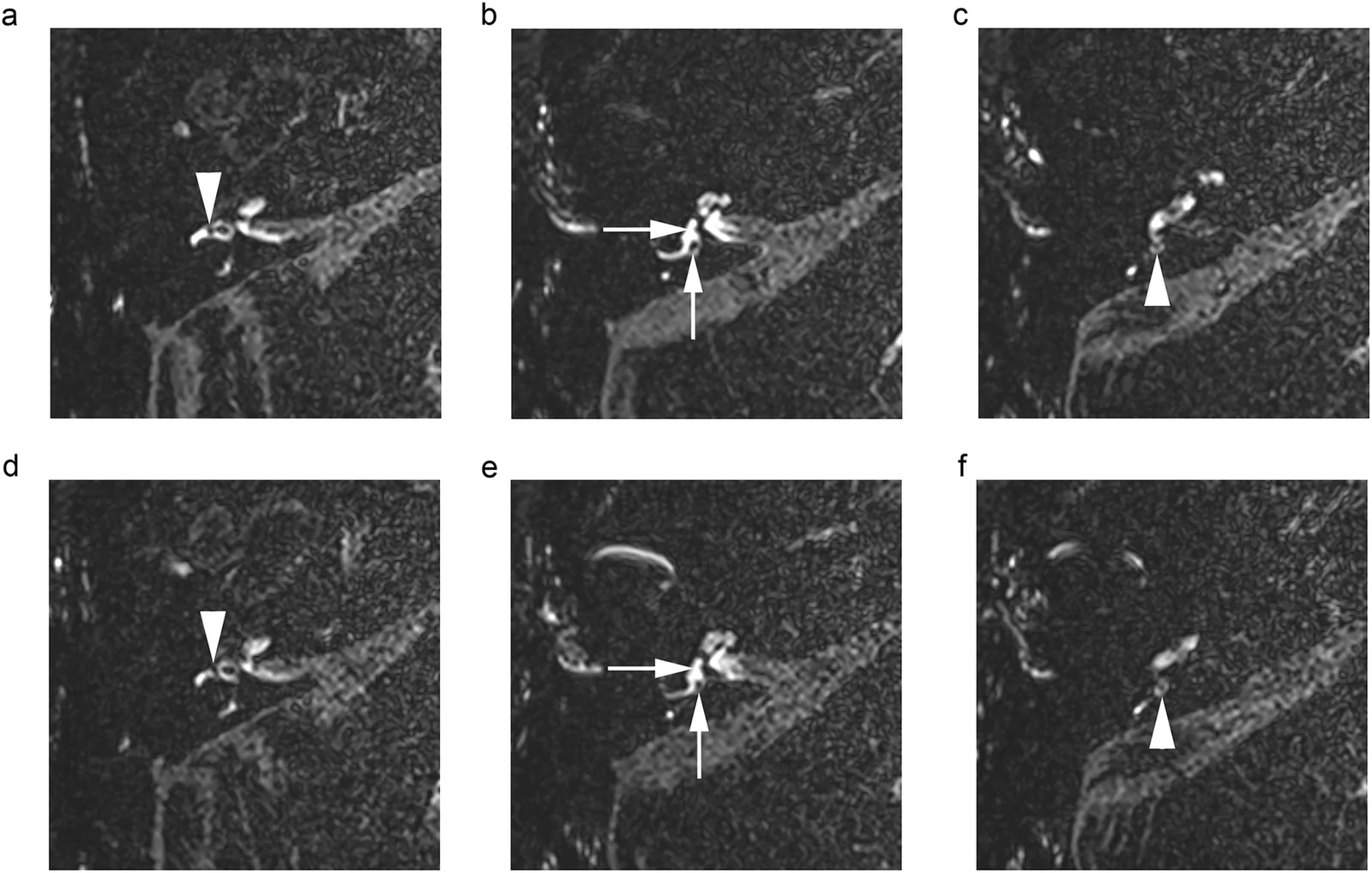
Comparable contrast resolution of half-dose gadopiclenol and full-dose gadobutrol on delayed hydrops MRI. A 42-year-old woman presented with vertigo, left-sided low-frequency sensorineural hearing loss, and tinnitus. Delayed 4-h post-Gd hydrops MRI was performed in August 2025 using a half-dose of gadopiclenol (0.05 mmol/kg; **a**–**c**) and repeated in June 2026 using a full dose of gadobutrol (0.1 mmol/kg; **d**–**f**) because of worsening symptoms and increasing attack frequency. The 2025 examination was normal, whereas the 2026 examination demonstrated increased PE and a ruptured saccule in the left ear, consistent with definite MD; these abnormal left-sided findings are not shown. Delayed post-Gd 3D-SPACE FLAIR images of the normal right inner ear from both examinations are displayed at matched anatomical levels from superior to inferior. The images show comparable PE and equivalent delineation of the endolymphatic spaces with half-dose gadopiclenol and full dose gadobutrol. **a**, **d** Both intra-individual examinations show similar PE of the lateral semicircular canal, with comparable visualisation of its ampulla (small arrowhead), a sensitive indicator of contrast resolution. **b**, **e** Both examinations show similar vestibular PE, with clear separate visualisation of the saccule and utricle. The saccule is located anteriorly along the medial vestibular wall (horizontal arrow), whereas the utricle is positioned posterosuperiorly within the vestibule (vertical arrow). **c**, **f** Both examinations show similar PE of the posterior semicircular canal, with sharp delineation of its ampulla (large arrowhead), again indicating comparable contrast resolution with both contrast agents

Gadopiclenol is a promising newer contrast agent for delayed inner-ear MRI because of its substantially higher longitudinal relaxivity. Its T1 relaxivity in human plasma/serum at 1.5 T and 37 °C is 12.8 L mmol⁻¹ s⁻¹, compared with 5.2 for gadobutrol, 4.1 for gadoteridol and gadopentetate dimeglumine, and 6.3 for gadobenate dimeglumine [38]. In the only hydrops-specific study published to date, half-dose IV gadopiclenol, 0.05 mmol/kg, at 3 T enabled hydrops detection that was clinically concordant with a standardised full-dose gadobenate protocol. SNR and CNR were comparable between protocols, and interobserver agreement for hydrops grading was excellent, with κ values greater than 0.80 [39]. Half-dose administration of high-relaxivity GBCAs may therefore help reduce Gd exposure, tissue retention, and environmental burden. Although preliminary, these findings are encouraging and support further evaluation of gadopiclenol for delayed inner-ear MRI (Fig. 11).

### Sequence-dependent optimisation of scan delay

Timing should be standardised together with the pulse sequence, because even small delay differences can alter perilymphatic signal and thereby affect the apparent degree of hydrops and enhancement. Earlier timing work showed that the optimal interval cannot be extrapolated from healthy volunteers alone, and the 2022 consensus established 4-h delayed IV imaging as the current standard [1, 34].

Two-hour imaging is promising but remains sequence-dependent rather than universally established. Early scanning may overestimate hydrops when perilymphatic filling is incomplete, whereas later imaging—typically 4 to 6 h—still provides higher signal and CNR. However, with optimised protocols, particularly gadobutrol-enhanced 3D-FLAIR, 2-h imaging may offer practical diagnostic equivalence [40, 41]. For 3D-real IR, the evidence is less consistent: 3.5 h appears feasible in some protocols, 3 h may be insufficient, and highly optimised T2-prepared ZOOMit SPACE real-IR may allow reliable 2-h imaging despite lower SNR/CNR than later scans [28, 42, 43]. Thus, the key question is not only “what delay?” but “what delay for which sequence?”

### Two-step or single-step protocol

**Fig. 12:**
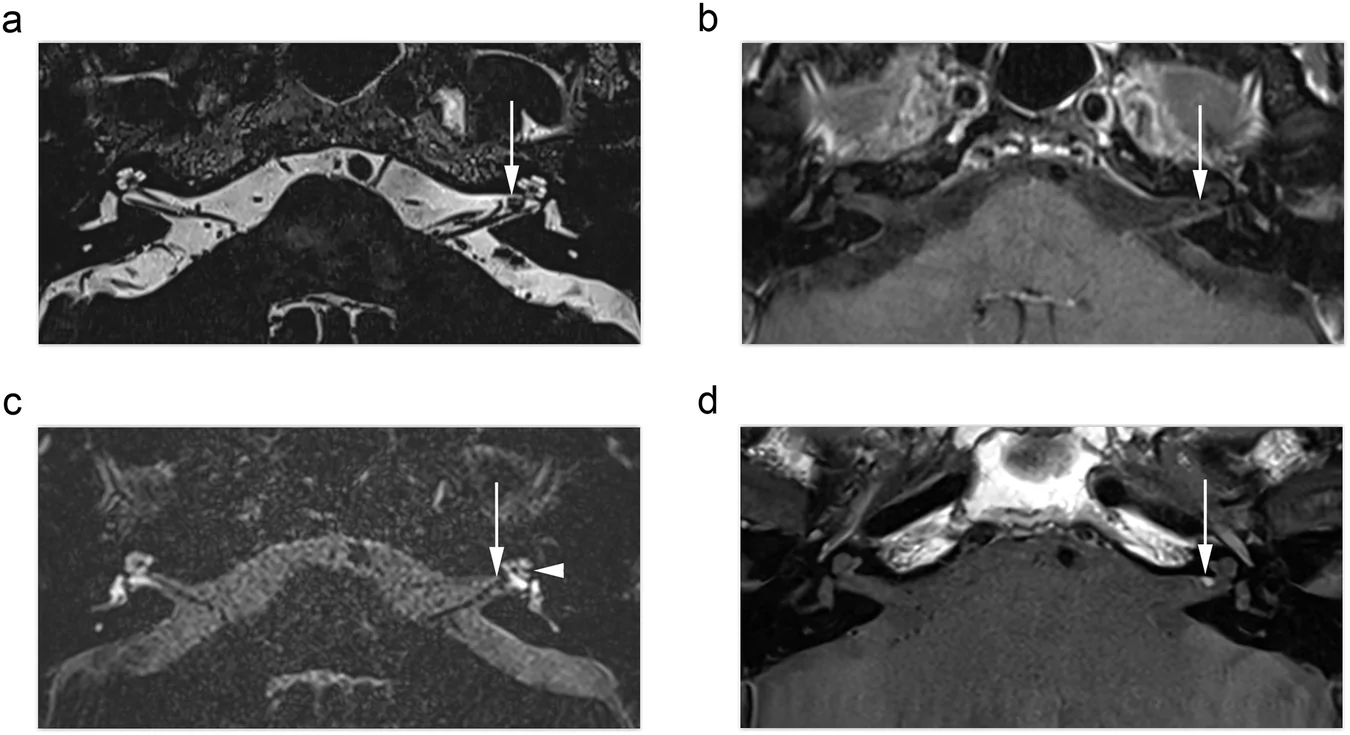
Added value of the two-step hydrops protocol in secondary hydropic ear disease. A 72-year-old woman presented with sudden left-sided sensorineural hearing loss and aural pressure. In the two-step protocol, conventional inner-ear sequences are obtained immediately after IV GBCA administration, followed by delayed hydrops imaging approximately 4 h after injection. **a** Axial heavily T2-weighted image through the internal auditory canal (IAC) at the midmodiolar level shows a small, sharply marginated hypointense nodular lesion in the left IAC, anterior to the vestibulocochlear nerve (vertical arrow). **b** Early post-Gd fat-suppressed T1-weighted image at the same level shows no enhancement of the lesion, making an intracanalicular schwannoma unlikely (vertical arrow). **c** Four-hour delayed post-Gd 3D-SPACE FLAIR image shows band-like hypointensity in the left middle cochlear turns, consistent with grade 2 cochlear EH according to the Bernaerts grading (arrowhead). The IAC lesion is barely visible on this delayed hydrops sequence, illustrating the limitation of a single-step protocol (vertical arrow). **d** An additional unenhanced, non-fat-suppressed T1-weighted image obtained the following day shows intrinsic T1 hyperintensity of the lesion, consistent with a small lipochoristoma of the internal auditory canal, formerly termed a lipoma (vertical arrow). The lesion was considered likely to impair inner-ear fluid dynamics by obstructing perilymphatic flow within the internal auditory canal, thereby contributing to secondary EH. This causative lesion is best identified on the conventional first step of the two-step imaging protocol

MRI for EH can be performed using either a two-step or single-step protocol (Table 2). In the two-step protocol, conventional posterior fossa and inner-ear sequences are acquired before and/or immediately after GBCA administration, followed by the dedicated hydrops sequence approximately 4 h after IV injection. This “double protocol” is mainly used for the initial evaluation of patients with suspected MD, when baseline imaging is needed to exclude alternative causes of audiovestibular symptoms, including small vestibular schwannoma, labyrinthitis, haemorrhage, congenital malformation, or other cerebellopontine angle and posterior fossa pathology (Fig. 12). It is particularly relevant in idiopathic sudden sensorineural hearing loss, where unenhanced and early post-contrast images help evaluate labyrinthitis and other acute inner-ear abnormalities [44]. However, whether all patients with SSNHL should undergo a complete EH MRI protocol remains uncertain, given the economic and logistical implications of occupying two scanner slots.

**Table 2:**
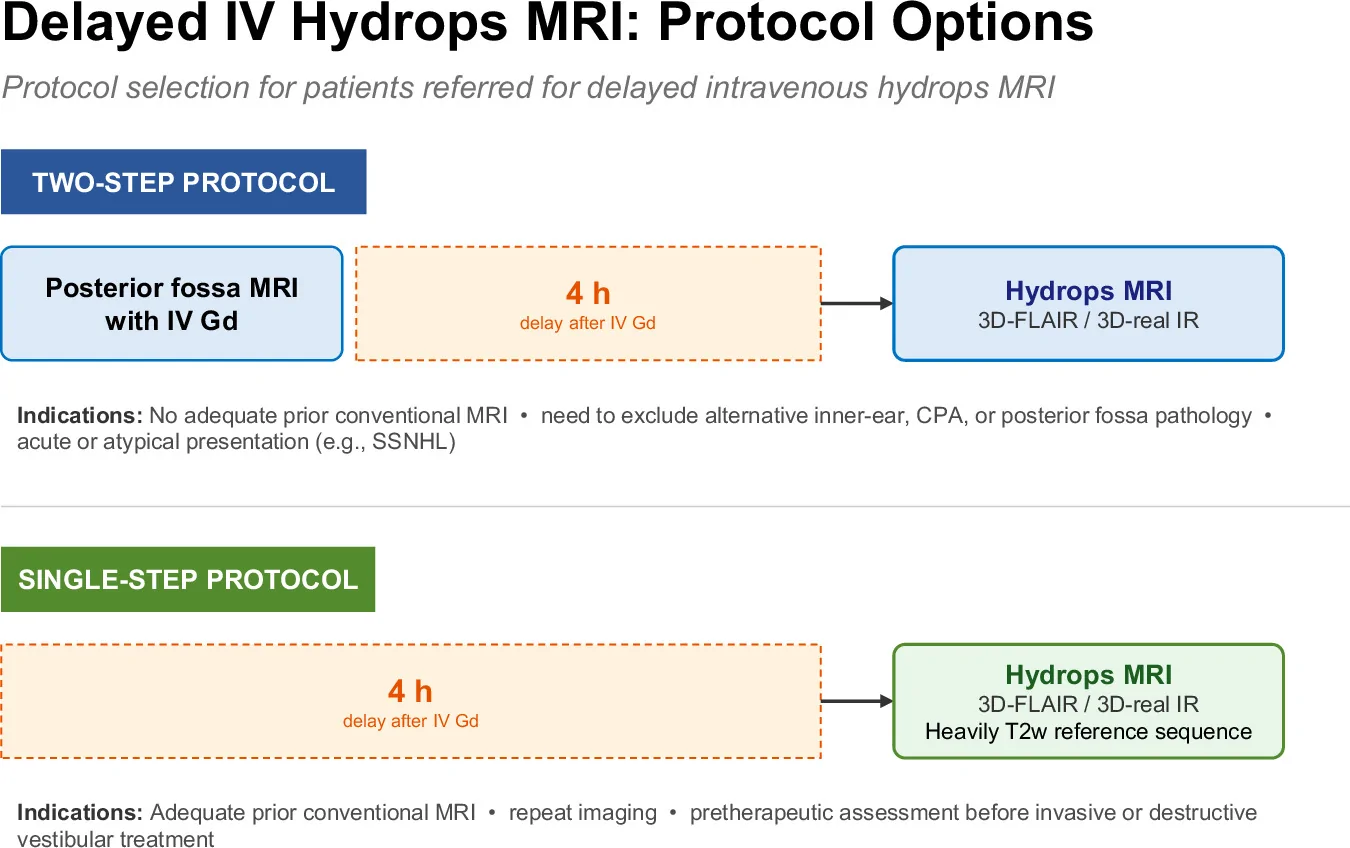
Single-step and two-step protocols for delayed hydrops MRI

Alternatively, hydrops MRI can be performed using a single-step protocol, in which the patient receives IV GBCA and returns approximately 4 h later for the delayed hydrops acquisition only. Because this approach does not include true pre-contrast or early post-contrast imaging, it is best reserved for patients who have already undergone conventional MRI of the posterior fossa and inner ear. The single-step protocol is particularly well suited for repeat hydrops imaging when symptoms or laterality change or after an equivocal or technically inadequate examination, for selected post-treatment monitoring, or before invasive or vestibular-destructive treatment. In the latter setting, assessment of the contralateral ear is clinically important, as contralateral hydrops may influence the decision to avoid a destructive procedure. In abbreviated protocols, a high-resolution heavily T2-weighted sequence may serve as an anatomical reference, as focal corresponding T2 signal loss may indicate intralabyrinthine schwannoma or fibrosis rather than hydrops [45]. The single-step protocol is more practical and resource-efficient, requiring only one scanner slot, while high-resolution heavily T2-weighted imaging can exclude most vestibular schwannomas and congenital malformations. However, very small enhancing lesions may occasionally be missed.

## Conclusion

Over the past 15 years, delayed IV Gd-enhanced MRI of EH has evolved from an experimental technique into a reliable diagnostic tool. Its performance is determined not by any single sequence parameter, but by the careful optimisation and interplay of multiple protocol components. A robust hydrops MRI protocol typically employs 3D-FLAIR or 3D-real IR sequences with a long echo train and prolonged TE to improve acquisition efficiency and background suppression, a long TR to enhance the perilymphatic signal, and a TI tailored to the selected TR to selectively null the endolymphatic-fluid signal.

Emerging deep-learning acceleration techniques may further improve clinical feasibility by shortening acquisition times or enabling ultra-high-resolution imaging with improved hydrops detection. In parallel, comparable diagnostic performance may be achievable with half-dose high-relaxivity agents, provided that these approaches are validated within the complete imaging protocol.

Continued standardisation and refinement of acquisition parameters, post-processing and contrast administration are essential to improve interpretation and reproducibility across centres. Because the key sequence parameters are highly interdependent, no single element should be modified in isolation without revalidating the entire protocol. In EH MRI, protocol design is therefore not merely technical optimisation; it is the foundation of diagnostic reliability.

## Data Availability

All data are available as figures.

## Abbreviations

3D-FLAIR: Three-dimensional fluid-attenuated inversion recovery
3D-real IR: Three-dimensional inversion recovery with real reconstruction
CNR: Contrast-to-noise ratio
CS: Compressed sensing
DLR: Deep learning reconstruction
EH: Endolymphatic hydrops
GBCA: Gadolinium-based contrast agent
Gd: Gadolinium
hT2w-3D-FLAIR: Heavily T2-weighted 3D-FLAIR
HYDROPS: Hybrid of reversed image of positive endolymph signal and native image of positive perilymph signal
IR: Inversion recovery
IV: Intravenous
MD: Ménière’s disease
MRI: Magnetic resonance imaging
Mxy: Transverse magnetization
Mz: Longitudinal magnetization
PE: Perilymphatic enhancement
PI: Parallel imaging
SNR: Signal-to-noise ratio
SPACE: Sampling perfection with application-optimised contrast using different flip angle evolution
TE: Time to echo
TI: Time to inversion
TR: Time to repetition
